# CO_2_ and aerosol concentration during manual and mechanical chest compression while cardiopulmonary resuscitation

**DOI:** 10.1097/MD.0000000000041528

**Published:** 2025-02-14

**Authors:** Johannes Heymer, Florian Dengler, Anna Hein, Alexander Krohn, Christina Jaki, Fabian Echterdiek, Stephan Schmid, Martina Müller-Schilling, Tobias Schilling, Matthias Ott

**Affiliations:** a Department of Interdisciplinary Emergency and Intensive Care Medicine, Klinikum Stuttgart, Stuttgart, Germany; b Simulation Center STUPS, Klinikum Stuttgart, Stuttgart, Germany; c Department of Internal Medicine, Klinikum Stuttgart, Stuttgart, Germany; d Department of Internal Medicine I, Gastroenterology, Hepatology, Endocrinology, Rheumatology and Infectious diseases, University Hospital Regensburg, Regensburg, Germany.

**Keywords:** aerosols, COVID-19, CPR, staff-to-staff-infection

## Abstract

**Background:**

This study investigates the staff-to-staff transmission risk of Coronavirus disease 2019 during cardiopulmonary resuscitation in an ambulance vehicle.

**Methods:**

Comparing manual and mechanical chest compressions, CO_2_ concentrations were monitored as a proxy for infection risk.

**Results:**

Results suggest that mechanical chest compressions generate lower CO_2_ levels, indicating a reduced risk of infection among healthcare workers compared to manual compressions.

**Conclusions:**

These findings highlight the potential benefits of employing mechanical chest compressions to mitigate staff-to-staff infections in small, confined spaces during aerosol-transmitted diseases.

## 1. Introduction

Severe acute respiratory syndrome Corona virus 2 (SARS-CoV-2) causes Coronavirus disease 2019 (COVID-19) and led to major losses of human life.^[1,2]^ There is mounting evidence that besides transmission via large droplets, SARS-CoV-2 is transmitted via inhalation of aerosols.^[1,3–12]^ Consistent with that, transmission is much easier indoors than outdoors.^[2,7,13,14]^ Studies have shown that aerosol emission and transmission risk increase during sports and indoor activities.^[15,16]^ Therefore, we suspect that performing chest compressions during patient care and training carries a high risk. Due to its distinct contagiousness, health care workers (HCW) are at high risk of infection with SARS-CoV-2 during patient care particularly in small spaces like ambulance vehicles.^[17–20]^

Not only does patient-to-patient transmission pose a significant risk, but staff-to-staff infection is particularly concerning in times of high prevalence of COVID-19 in the population.^[21]^ With an increasing number of affected employees, there is a risk that for example the prehospital emergency medicine system may no longer be guaranteed.^[22–24]^

Direct and real time measurement of virus containing aerosols is difficult. Since CO_2_ is co-exhaled with aerosols containing SARS-CoV-2 its concentration can be used as a proxy of respiratory infectious disease transmission risk and SARS-CoV-2 concentration indoors.^[2,25,26]^ Low-cost CO_2_ sensors have become suitable for indicating indoor aerosol transmission risk in different settings like school class rooms.^[2,8,25,27,28]^ Chest compression during cardiopulmonary resuscitation (CPR) can either be performed manually or with mechanical chest compression devices.

This study aimed to figure out, whether CO_2_ concentration increases during indoor chest compressions. To assess whether manual chest compressions increase the risk of infection, we compared the results with simulated patient care without manual chest compressions with the meaning of using a mechanical chest compression device. This may be relevant for staff safety and infection prevention during patient care with aerosol transmitted diseases.

## 2. Materials and methods

**Figure 1.**
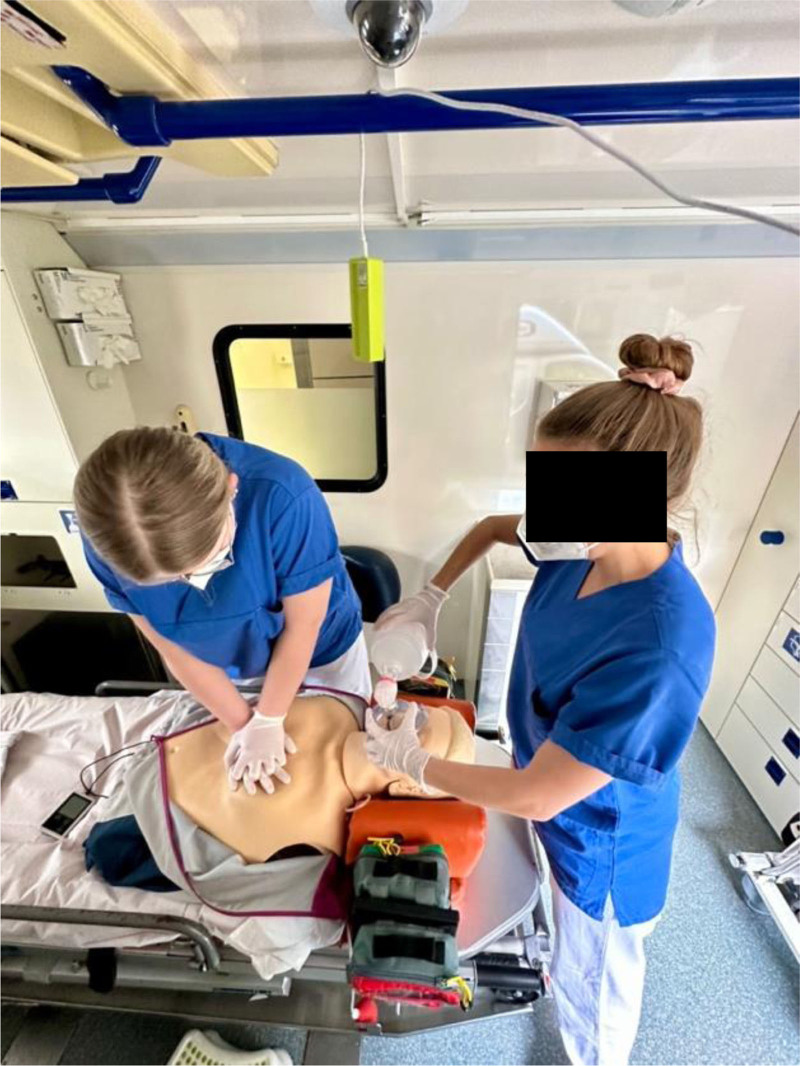
Study design in the ambulance car.

We performed CPR in a standard German ambulance vehicle (System Strobel GmbH & Co. KG, Aalen, Germany) which metrics are approximately 3.45 m length, 2.0 m width, and 1.95 m height. Seven groups consisting of 2 health care professionals performed chest compressions and bag-mask ventilation 30:2 in the ambulance vehicle for 7 minutes on a CPR manikin in the first instance. Secondly, they did not perform manual chest compressions to simulate ongoing mechanical chest compressions by the compression device. They were allowed to talk to each other, and each window of the vehicle was closed. All professionals were wearing N95 masks. We monitored CO_2_ concentrations using a low-budget CO_2_ sensor (TFA-Dostmann Airco2ntrol Mini, TFA Dostmann GmbH & Co. KG, Wertheim-Reicholzheim, Germany), which was hanging above the CPR manikin on the ceiling of the ambulance vehicle. Between each round, the ambulance car was well ventilated by opening the doors and windows until measured CO_2_ concentration was at a baseline again. The design of the study in the ambulance car is illustrated in Figure 1. An analysis of this kind does not require ethical approval according to our regulations. However, these settings are approved by the works council of our hospital (Klinikum Stuttgart, Katharinenhospital, Stuttgart, Germany).

### 2.1. Statistical methods

To test for normal distribution the Kolmogorov–Smirnov test was used with a *P* value < .05. Normal distributed data were described with mean and standard deviation. We used GraphPad PRISM for Windows (GraphPad Software Inc, Boston) software to create graphs.

Due to the small sample size with 7 groups for manual and mechanical CPR we used a Bayesian approach to analyze our data. We compared the CO_2_ concentration before and after CPR was performed for both groups and we compared the CO_2_ concentration after manual versus mechanical CPR was performed. To decide whether there was a credible difference between the CO_2_ concentrations we applied the method of using the high density interval (HDI) and region of practical equivalence (ROPE) as described by Kruschke.^[29,30]^

We used the European norm for indoor air quality EN 13779: 2007^[31]^ to set the value for the ROPE. This norm for indoor air quality defines 4 categories: IDA 1: <800, IDA 2: 800 to 1000, IDA 3: 1000 to 1400, and IDA 4: >1400 ppm. We therefore set our region of practical equivalence with a width of 400 ppm.

## 3. Results

We recruited medical professionals with averaged 175 ± 10 cm height and 71 ± 15 kg body weight and consequential a body mass index of 20.2 ± 3.7 kg/m^2^.

All values for the CO_2_ concentrations were normal distributed. The 95% HDI for the difference of means before and after manual and mechanical CPR excluded zero and was outside the ROPE. We are therefore confident, that the CO_2_ concentrations in an emergency ambulance increase during a 7-minute period of mechanical or manual CPR.

CO_2_ level amounted 484.6 (±59.5) before and 1216 (±183.1) ppm after manual CPR with a difference of means of 732 what was considered a credible difference.

In comparison, CO_2_ level amounted 413.3 (±38.2) before mechanical CPR and 799.9 (±69.3) ppm after mechanical CPR with a difference of means of 389, what also was considered a credible difference.

Furthermore, we found a credible difference between the CO_2_ values after manual CPR with 1216 (±183.1) versus after mechanical CPR with 799.9 (±69.3) ppm with a difference of means of 417 (210, 633; 95% HDI).

This result provides confidence, that CO_2_ values are higher after manual CPR compared to mechanical CPR. Our wide ROPE of 400 ppm, which corresponds the wide of an indoor air quality category as defined by the EN 13779: 2007^[31]^ provides additional confidence regarding the difference between the measured CO_2_ concentrations.

**Figure 2.**
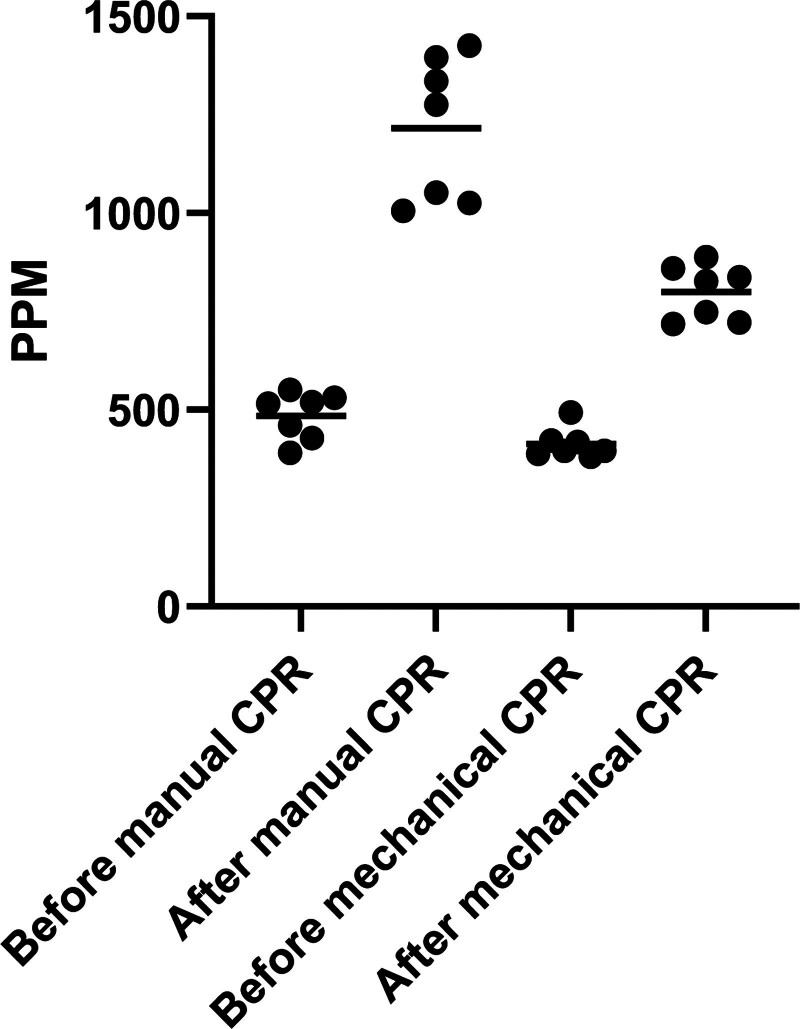
CO_2_ concentrations in parts per million (ppm) before and after manual or mechanical CPR. CPR = cardiopulmonary resuscitation, ppm = parts per million.

The datasets can be found in detail in Table 1. The individual data points have been illustrated in Figure 2 for graphical comparison between manual and mechanical before and after CPR, respectively.

**Table 1:** Data set for the statistical analysis.

	Before manual CPR (mean [SD])	After manual CPR (mean [SD])	Difference of means (95% HDI)	ROPE width	Decision
CO_2_ level (ppm)	484.6 (59.5)	1216 (183.1)	732 (534, 927)	400	Credible difference
Normal distribution	Yes	Yes			
	Before mechanical CPR (mean [SD])	After mechanical CPR (mean [SD])	Difference of means (95% HDI)	ROPE width	Decision
CO_2_ level (ppm)	413.3 (38.2)	799.9 (69.3)	389 (306, 472)	400	Credible difference
Normal distribution	Yes	Yes			
	After manual CPR (mean [SD])	After mechanical CPR (mean [SD])	Difference of means (95% HDI)	ROPE width	Decision
CO_2_ level (ppm)	1216 (183.1)	799.9 (69.3)	417 (210, 633)	400	Credible difference
Normal distribution	Yes	Yes			

CPR = cardiopulmonary resuscitation, HDI = high density interval, PPM = parts per million, ROPE = region of practical equivalence, SD = standard deviation.

## 4. Discussion

Since there is overwhelming evidence that SARS-CoV-2 is transmitted via aerosols^[5,7–12,32]^ and CO_2_ is co-exhaled,^[2]^ experts agree that CO_2_ concentrations can be used as a proxy for virus concentrations indoors.^[2]^ The use of low-cost CO_2_ sensors is prevalent because background CO_2_ level is almost stable and raising CO_2_ concentrations indoors is usually only from human exhalation.^[2,27,28]^ Because evidence is suggesting that SARS-CoV-2 is spread primarily via indoor airborne transmission,^[8]^ we wanted to investigate CO_2_ concentrations during patient care in an ambulance car which is relatively small. The ambulance car was well-ventilated before each experiment. Measured CO_2_ concentrations before mechanical or manual chest compressions were in the range of outdoor CO_2_ concentrations, which are typically between 350 and 450 ppm.^[8]^ After manual or mechanical chest compressions, the concentrations raised to a credible difference. The World Health Organization declared a limit of 1000 ppm CO_2_ for prevention of COVID-19 transmission, furthermore the indoor CO_2_ concentrations in classrooms should not exceed 700 ppm, which are much bigger than an ambulance car cabin.^[1]^ The concentrations after manual chest compressions exceeded the limit of 1000 ppm with 1216 (±183.1), the concentrations after mechanical chest compressions exceeded 700 ppm with 799.9 (±69.3). Our study suggests that resuscitation efforts by 2 health care professionals in an ambulance car led to CO_2_ concentrations which indicate a risk of staff-to-staff infection. In HCW infections, staff to staff transmission poses a significant challenge. For instance, Gordon et al^[21]^ discovered that 79% of local HCW COVID-19 infections occurred due to exposure to other staff. The results of our investigation show that the concentrations of CO_2_ are lower if chest compressions are performed mechanically by a chest compression device. This may be attributed to reduced physical efforts and thus less endogenous CO_2_ production and less respiratory minute ventilation.

Based on these findings, we conclude that personal protective equipment is crucial during training and patient care to reduce the risk of infection, even if the patient is not infectious. Personal protective equipment does not offer perfect protection. From personal experience, masks often slip, become less effective when wet with sweat, or are pulled down due to exhaustion during patient care. Possibly, mechanical chest compression devices therefore provide additional protection regarding staff-to-staff infection.

The present study suggests that there is an extremely high risk of infection for providers, as previously mentioned in the studies cited. Cardiopulmonary resuscitations occur in both clinical and preclinical settings, and our results can be applied to other conditions. Our work is particularly important because it simultaneously offers a proposal to mitigate the risk through performing mechanical CPR. Therefore, this study aims to raise awareness about provider-to-provider infections and, at the same time, provide a solution by using mechanical CPR. Furthermore, a recent systematic review and meta-analysis indicates that mechanical chest compression is noninferior to manual chest compressions.^[33]^ The limitation of our study is the small sample size.

## 5. Conclusion

In times of COVID-19 or other aerosol transmitted diseases, the application of mechanical chest compressions instead of performing manual chest compressions during resuscitation in small rooms may reduce the risk of staff-to-staff infection. Our study suggests, that CO_2_ concentrations during patient care in an ambulance car are raising to a dangerous amount according infection risk during few minutes.

## Author contributions

**Conceptualization:** Johannes Heymer, Florian Dengler, Alexander Krohn, Matthias Ott.

**Data curation:** Florian Dengler.

**Formal analysis:** Johannes Heymer, Anna Hein, Fabian Echterdiek, Matthias Ott.

**Investigation:** Johannes Heymer, Florian Dengler, Christina Jaki, Matthias Ott.

**Methodology:** Johannes Heymer, Florian Dengler, Christina Jaki, Stephan Schmid, Matthias Ott.

**Project administration:** Johannes Heymer, Matthias Ott.

**Resources:** Christina Jaki.

**Supervision:** Johannes Heymer, Tobias Schilling.

**Validation:** Martina Müller-Schilling.

**Visualization:** Alexander Krohn, Matthias Ott.

**Writing – original draft:** Matthias Ott.

**Writing – review & editing:** Johannes Heymer, Florian Dengler, Anna Hein, Alexander Krohn, Christina Jaki, Fabian Echterdiek, Stephan Schmid, Martina Müller-Schilling, Tobias Schilling, Matthias Ott.
